# Association of Coronary Microvascular Dysfunction With Cerebrovascular Risk and Cognitive Decline in Patients With Type 2 Diabetes Mellitus: A Cross-Sectional Study

**DOI:** 10.7759/cureus.110172

**Published:** 2026-06-03

**Authors:** Adnan Imran, Kamran Memon, Hafiz Abdul Waris, Sikander Khan Tanoli, Kainaat Nawaz Jadoon, Azeem Gohar Khan, Muhammad Moazzam, Hassan Saeed

**Affiliations:** 1 Department of Acute Medicine, Walsall Manor Hospital, Walsall, GBR; 2 Department of Medicine, Isra University Hospital Hyderabad, Hyderabad, PAK; 3 Department of Cardiology, Walsall Manor Hospital, Walsall, GBR; 4 Department of Acute Medicine, University Hospitals Coventry and Warwickshire, Warwickshire, GBR; 5 Department of Obstetrics and Gynecology, Ayub Teaching Hospital, Abbottabad, PAK; 6 Department of Cardiology, Jinnah International Hospital, Abbottabad, PAK; 7 Department of Internal Medicine, Wapda Hospital Complex, Lahore, PAK; 8 Department of Cardiac Emergency, Armed Forces Institute of Cardiology and National Institute of Heart Diseases, Lahore, PAK

**Keywords:** cardiology research, cerebrovascular, cognitive decline, coronary intervention, coronary microvascular dysfunction, diabetes, microvascular, type 2 diabetes mellitus

## Abstract

Background: Type 2 diabetes mellitus is associated with microvascular complications that may contribute to both cardiovascular and neurological morbidity.

Objective: To evaluate the association of coronary microvascular dysfunction with stroke risk, cognitive decline, and selected hematologic/metabolic biomarkers in patients with type 2 diabetes mellitus without major vessel occlusion.

Methods: This analytical cross-sectional study was conducted at the Department of Cardiology, Jinnah International Hospital, Abbottabad, Pakistan, from May 2025 to November 2025. A total of 255 patients with type 2 diabetes mellitus were enrolled using non-probability consecutive sampling. Coronary microvascular dysfunction was assessed through clinical evaluation and available cardiovascular investigations in patients without significant epicardial coronary artery obstruction.

Results: The mean age of the participants was 54.7 ± 10.9 years, and 146 (57.3%) were male. Coronary microvascular dysfunction was identified in 112 (43.9%) patients. Cognitive decline was significantly more frequent among patients with coronary microvascular dysfunction than those without it (46.4% vs. 29.4%, p = 0.006). Elevated stroke risk was also higher in patients with coronary microvascular dysfunction (43.8% vs. 23.1%, p = 0.001). Patients with coronary microvascular dysfunction had longer diabetes duration (10.1 ± 4.3 vs. 7.4 ± 3.6 years, p = 0.001), higher glycosylated hemoglobin levels (8.9 ± 1.6% vs. 7.8 ± 1.4%, p = 0.001), higher low-density lipoprotein cholesterol levels (142.1 ± 27.5 vs. 128.4 ± 28.7 mg/dL, p = 0.004), and greater frequencies of hypertension (63.4% vs. 46.9%, p = 0.012) and dyslipidemia (55.4% vs. 41.3%, p = 0.028).

Conclusion: Coronary microvascular dysfunction was common among patients with type 2 diabetes mellitus and was significantly associated with cognitive decline, elevated stroke risk, and adverse metabolic profiles. Early identification of microvascular dysfunction may improve vascular risk stratification and guide preventive strategies in diabetic patients.

## Introduction

Diabetes mellitus (DM) is a common and serious worldwide problem and is a major risk factor for cardiovascular and cerebrovascular disease [1]. While there has been a significant emphasis on the role of macrovascular pathology such as coronary artery stenosis and stroke, there is emerging evidence that microvascular dysfunction also plays a significant role in the pathogenesis of poor clinical outcomes in patients with diabetes [2]. One such complication is coronary microvascular dysfunction, which is an important but often overlooked condition that can occur in the presence or absence of severe epicardial coronary artery stenosis [3]. Coronary microvascular dysfunction is defined as dysfunction in the regulation of blood flow in the small coronary arterioles and capillaries that deliver blood to the myocardium [4]. This condition involves the small vessels and may not be diagnosed on conventional angiography since the larger coronary vessels are generally clear or non-obstructed [5]. In type 2 diabetes, chronic exposure to hyperglycemia, insulin resistance, oxidative stress, endothelial dysfunction, low-level inflammation, and advanced glycation end products (AGEs) play a role in structural and functional changes in the microvasculature [6]. These changes lead to dysfunctional vasodilation, decreased coronary flow reserve, and reduced tissue perfusion, which in turn affect cardiac function despite the absence of large-vessel obstructive disease [7].

Coronary microvascular dysfunction is not only relevant in terms of heart function. Recent work has shown that microvascular disease may be a systemic vascular disease that affects more than one organ, such as the brain [8]. In patients with diabetes, the same pathological processes that are responsible for coronary microvascular dysfunction may also affect cerebral microvessels, leading to ischemic brain damage, silent brain infarction, white matter lesions, and progressive cognitive impairment [9]. This suggests that coronary microvascular dysfunction may be a marker of systemic vascular damage and may predict stroke and neurocognitive dysfunction [10,11]. Type 2 diabetes is associated with a high risk of stroke and disability [12]. Though large-vessel atherosclerosis is well known, cerebral microvascular dysfunction is emerging as a significant cause of symptomatic and asymptomatic cerebrovascular disease [13]. Likewise, the mechanisms underlying cognitive impairment in diabetes are complex and may be due to chronic vascular dysfunction, intermittent subclinical ischemia, inflammation, and metabolic dysfunction [14,15]. Given the similar underlying mechanisms of the coronary and cerebral microvasculatures, investigating coronary microvascular dysfunction may help better understand the risk of stroke and neurocognitive impairment in diabetic patients. Therefore, this study was conducted to address this gap by evaluating the association of coronary microvascular dysfunction with stroke risk, cognitive decline, and hematologic/metabolic biomarkers in patients with type 2 diabetes mellitus without evidence of major coronary or cerebral vessel occlusion.

## Materials and methods

This analytical cross-sectional study was conducted at the Department of Cardiology, Jinnah International Hospital, Abbottabad, Pakistan, from May 2025 to November 2025. A total of 255 patients were included using a non-probability consecutive sampling technique. A non-probability consecutive sampling technique was used because it allowed feasible enrollment of all eligible patients presenting during the study period. However, this approach may introduce selection bias, and this limitation has been acknowledged. Inclusion and exclusion criteria are presented in Table 1.

**Table 1:** Inclusion and exclusion criteria. * Clinically stable patients were defined as those without acute coronary syndrome, acute chest pain requiring emergency intervention, hemodynamic instability, acute neurological deficits, active infection, or hospitalization for a major medical event at the time of enrollment.

Inclusion criteria	Exclusion criteria
Patients aged 30-75 years with confirmed type 2 diabetes mellitus	Known obstructive coronary artery disease
Patients without evidence of major coronary vessel occlusion on clinical evaluation or imaging	Previous myocardial infarction
Patients without evidence of major cerebral vessel occlusion on clinical evaluation or imaging	History of coronary revascularization
Clinically stable patients*	Significant valvular heart disease
Patients willing to participate and provide informed consent	History of major stroke
Patients with complete clinical and laboratory data available	Neurological disorders affecting cognitive assessment (e.g., dementia, Alzheimer's disease, Parkinson's disease, multiple sclerosis, epilepsy requiring ongoing treatment, brain tumors, traumatic brain injury with persistent cognitive impairment, or other neurodegenerative disorders)
	Advanced chronic kidney disease (stage 4-5)
	Active infection or inflammatory disease
	Current malignancy or undergoing cancer treatment
	Other systemic illnesses significantly affecting cardiovascular or neurological status

Data collection

After obtaining approval from the institutional review board and the ethical committee, patients fulfilling the inclusion criteria were enrolled after obtaining informed consent. Demographic and clinical information, including age, gender, duration of diabetes, medical history, and medication use, was recorded. Clinical examination included measurement of blood pressure, heart rate, and body mass index. Evaluation of coronary microvascular dysfunction was based on clinical assessment and available cardiovascular investigations in patients without significant epicardial coronary artery obstruction. Stroke risk was assessed using clinical history, neurological evaluation, and documented vascular risk factors, such as hypertension, dyslipidemia, and duration of diabetes. Coronary microvascular dysfunction was defined as evidence of impaired coronary microvascular perfusion in the absence of obstructive epicardial coronary artery disease. Diagnosis was based on available cardiovascular investigations, including non-invasive ischemia assessment and documentation of reduced coronary flow reserve where available. Patients with significant epicardial coronary artery stenosis were excluded. In cases where advanced coronary microvascular testing such as PET, cardiac MRI perfusion imaging, or invasive coronary function testing was not available, the diagnosis was made using clinical and available institutional assessment, and this limitation was acknowledged. Cognitive status was assessed through general clinical neurological evaluation focusing on memory, attention, orientation, and daily functioning, as recorded in the patient’s clinical examination notes, without the use of licensed cognitive assessment tools or proprietary questionnaires. Cognitive decline was assessed through general clinical neurological evaluation of memory, attention, orientation, and daily functioning. No standardized cognitive assessment tool, such as Montreal Cognitive Assessment (MoCA) or Mini-Mental State Examination (MMSE), was used. Blood samples were collected from all participants to analyze laboratory parameters, including fasting blood glucose, glycosylated hemoglobin (HbA1c), lipid profile, complete blood count, renal function tests, and other routine biochemical markers associated with vascular health. These laboratory values were evaluated to determine their relationship with coronary microvascular dysfunction and neurological outcomes.

Data analysis procedure

All collected data were entered and analyzed using IBM SPSS version 26.0 (IBM Corp., Armonk, NY). Quantitative variables were presented as mean ± standard deviation and qualitative variables as frequencies and percentages. Normality was assessed using the Shapiro-Wilk test. An independent samples t-test was used for comparison of continuous variables, while a chi-square test was applied for categorical variables. Where appropriate, 95% confidence intervals were calculated for key outcome measures. Multivariate logistic regression analysis was considered to identify independent associations of coronary microvascular dysfunction with cognitive decline and elevated stroke risk after adjusting for potential confounders, including age, gender, duration of diabetes, hypertension, dyslipidemia, HbA1c, and low-density lipoprotein (LDL) cholesterol. A p-value ≤ 0.05 was considered statistically significant.

## Results

The mean age of the patients with type 2 diabetes was 54.7 ± 10.9 years. The largest proportion of participants belonged to the 46-60 years age group (112, 43.9%), followed by those older than 60 years (75, 29.4%) and 30-45 years (68, 26.7%). Males constituted 146 patients (57.3%), while females accounted for 109 (42.7%). The mean duration of diabetes was 8.6 ± 4.2 years. Comorbid conditions were common, with hypertension present in 138 patients (54.1%) and dyslipidemia in 121 patients (47.5%) (Table 2).

**Table 2:** Demographic and clinical characteristics of the patients (n = 255).

Variable	Category	n (%)/Mean ± SD
Age (years)	—	54.7 ± 10.9
Age group	30-45 years	68 (26.7%)
	46-60 years	112 (43.9%)
	>60 years	75 (29.4%)
Gender	Male	146 (57.3%)
	Female	109 (42.7%)
Duration of diabetes (years)	—	8.6 ± 4.2
Hypertension	Yes	138 (54.1%)
	No	117 (45.9%)
Dyslipidemia	Yes	121 (47.5%)
	No	134 (52.5%)

Coronary microvascular dysfunction was identified in 112 patients (43.9%), while 143 patients (56.1%) did not have this condition. Cognitive decline was observed in 94 patients (36.9%), whereas 161 patients (63.1%) showed no evidence of cognitive impairment. Regarding stroke risk, 82 patients (32.2%) were classified as having an elevated risk, while the majority, 173 patients (67.8%), were categorized as having a lower risk (Table 3).

**Table 3:** Prevalence of coronary microvascular dysfunction, stroke risk, and cognitive decline (n = 255). Stroke risk was categorized based on predefined clinical and vascular risk factors, including age, hypertension, duration of diabetes, dyslipidemia, smoking status, and neurological history. A validated stroke risk score was not used, which has been acknowledged as a limitation.

Variable	Category	n (%)
Coronary microvascular dysfunction	Present	112 (43.9%)
	Absent	143 (56.1%)
Cognitive decline	Present	94 (36.9%)
	Absent	161 (63.1%)
Stroke risk	Elevated risk	82 (32.2%)
	Lower risk	173 (67.8%)

The metabolic profile of the participants indicated poor glycemic and lipid control. The mean fasting blood glucose level was 167.8 ± 46.3 mg/dL, and the average HbA1c was 8.3 ± 1.7%, reflecting suboptimal long-term glycemic control. Lipid parameters showed a mean LDL cholesterol of 134.5 ± 29.2 mg/dL and total cholesterol of 208.4 ± 37.6 mg/dL. The mean high-density lipoprotein (HDL) cholesterol was relatively low at 39.6 ± 7.8 mg/dL, while triglyceride levels averaged 186.7 ± 64.2 mg/dL (Table 4).

**Table 4:** Blood parameters of study participants (n = 255).

Variable	Mean ± SD
Fasting blood glucose (mg/dL)	167.8 ± 46.3
Glycosylated hemoglobin (HbA1c) (%)	8.3 ± 1.7
Low-density lipoprotein (LDL) cholesterol (mg/dL)	134.5 ± 29.2
High-density lipoprotein (HDL) cholesterol (mg/dL)	39.6 ± 7.8
Total cholesterol (mg/dL)	208.4 ± 37.6
Triglycerides (mg/dL)	186.7 ± 64.2

Coronary microvascular dysfunction showed a significant association with adverse neurological outcomes. Cognitive decline was more common among patients with coronary microvascular dysfunction (52, 46.4%) compared to those without it (42, 29.4%), and this difference was statistically significant (p = 0.006). Similarly, elevated stroke risk was observed in 49 patients (43.8%) with coronary microvascular dysfunction compared with 33 patients (23.1%) without it (p = 0.001), suggesting that coronary microvascular impairment may be linked with higher neurological risk (Table 5).

**Table 5:** Association of coronary microvascular dysfunction (CMD) with clinical outcomes (n = 255).

Variable	CMD present (n = 112), n (%)	CMD absent (n = 143), n (%)	χ² (df)	p-value	Effect size
Cognitive decline	52 (46.4%)	42 (29.4%)	7.85 (1)	0.006	Phi = 0.18
No cognitive decline	60 (53.6%)	101 (70.6%)			
Elevated stroke risk	49 (43.8%)	33 (23.1%)	12.30 (1)	0.001	Phi = 0.22
Lower stroke risk	63 (56.2%)	110 (76.9%)			

Several metabolic and clinical factors were significantly associated with coronary microvascular dysfunction. Patients with the condition had a longer duration of diabetes (10.1 ± 4.3 years) compared with those without it (7.4 ± 3.6 years, p = 0.001). Glycemic control was poorer in the affected group with a higher HbA1c level (8.9 ± 1.6%) compared to 7.8 ± 1.4% in patients without dysfunction (p = 0.001). LDL cholesterol levels were also higher in the coronary microvascular dysfunction group (142.1 ± 27.5 mg/dL) compared with 128.4 ± 28.7 mg/dL (p = 0.004). Additionally, hypertension (63.4% vs. 46.9%, p = 0.012) and dyslipidemia (55.4% vs 41.3%, p = 0.028) were more frequent among patients with coronary microvascular dysfunction (Table 6).

**Table 6:** Association of clinical and metabolic factors with coronary microvascular dysfunction (CMD) (n = 255).

Variable	CMD present (Mean ± SD/n, %)	CMD absent (Mean ± SD/n, %)	Test statistic	p-value	Effect size
Duration of diabetes (years)	10.1 ± 4.3	7.4 ± 3.6	—	0.001	—
Glycosylated hemoglobin (%)	8.9 ± 1.6	7.8 ± 1.4	—	0.001	—
Low-density lipoprotein cholesterol (mg/dL)	142.1 ± 27.5	128.4 ± 28.7	—	0.004	—
Hypertension	71 (63.4%)	67 (46.9%)	χ² = 6.92, df = 1	0.012	Phi = 0.16
Dyslipidemia	62 (55.4%)	59 (41.3%)	χ² = 5.01, df = 1	0.028	Phi = 0.14

## Discussion

This study assessed the presence of coronary microvascular dysfunction and its relationship with stroke risk, cognitive impairment, and laboratory measures in patients with type 2 diabetes mellitus without major epicardial coronary artery obstruction. The results showed that a substantial number of patients with diabetes had coronary microvascular dysfunction, and this was strongly related to poor neurological events and metabolic dysfunction. The findings confirm the emerging view of microvascular disease in diabetes as a vascular process that involves multiple organs, such as the heart and brain [16]. In the current study, we found that 43.9% of type 2 diabetes mellitus patients had coronary microvascular dysfunction. This high incidence underlines the role of microvascular dysfunction in the development of cardiovascular disease in diabetes, even when the major epicardial coronary arteries are not severely blocked. Various mechanisms, such as chronic hyperglycemia, insulin resistance, oxidative stress, and endothelial dysfunction, are involved in the structural and functional changes of the coronary microvasculature. These microvascular changes result in impaired coronary flow reserve and impaired myocardial perfusion and are associated with an increased risk of cardiovascular complications despite unobstructed epicardial coronary arteries [17]. The link between coronary microvascular dysfunction and cognitive impairment found in this study supports the idea of common microvascular dysfunction between the heart and brain. Those with coronary microvascular dysfunction had a significantly greater incidence of cognitive impairment than those without microvascular dysfunction. Microangiopathy in diabetes may involve small cerebral vessels, resulting in chronic cerebral hypoperfusion, silent cerebral ischemia, and ensuing neuronal damage. These vascular abnormalities can result in cognitive dysfunction, memory impairment, and executive dysfunction in patients with diabetes [18].

In line with this, the current study found that patients with coronary microvascular dysfunction showed a significant increase in high stroke risk. It is well known that diabetes is a major risk factor for both ischemic and hemorrhagic stroke, and microvascular disease is also a significant factor in small vessel disease. The association between coronary microvascular dysfunction and stroke risk observed in this study suggests that microvascular abnormalities in the coronary arteries may reflect a widespread vascular dysfunction. Thus, this type of patient may be important for early risk assessment and development of strategies for preventing stroke [19]. Laboratory and metabolic factors were also significantly related to coronary microvascular dysfunction in our study. Fasting glucose and HbA1c levels were higher in the microvascular dysfunction group than in the group without microvascular dysfunction, suggesting worse glucose control. Hyperglycemia has been reported to induce endothelial dysfunction, oxidative stress, and inflammation, which damage the microcirculation. In addition, higher LDL cholesterol levels and the presence of dyslipidemia were also observed in patients with coronary microvascular dysfunction. Dyslipidemia may promote vascular injury by promoting the deposition of lipids, inflammatory response, and endothelial dysfunction [20]. Furthermore, this study also found an association between the duration of diabetes and coronary microvascular dysfunction. Chronic exposure to elevated glucose levels may result in microvascular damage, leading to structural changes (such as thickening of the capillary basement membrane, endothelial dysfunction, and decreased vascular compliance). This could potentially affect microvascular blood flow in the myocardium and brain, and hence contribute to cardiovascular diseases and brain dysfunction.

Patients with coronary microvascular dysfunction also had a higher incidence of hypertension. High blood pressure can also contribute to further endothelial damage and stiffening of the arteries, which impairs microvascular blood flow. The presence of diabetes combined with hypertension can be particularly harmful to the vascular system and may accelerate the development of microvascular disease in various organs [21]. Our study highlights the significance of considering coronary microvascular dysfunction as a possible marker for systemic vascular disease in patients with type 2 diabetes mellitus. Recognizing microvascular dysfunction early may allow clinicians to better identify patients at risk of stroke and dementia, even without blockage of major vessels. This may lead to earlier intervention with preventative measures such as better diabetes control, intensive cardiovascular risk factor management, and frequent neurological monitoring. Moreover, the findings of this study suggest routine blood tests, such as HbA1c and cholesterol, may be used as proxy measures of microvascular dysfunction. This suggests that improved metabolic control may help to minimize microvascular damage and prevent progression of both cardiovascular and neurological disease in diabetes.

Limitations

This study has several limitations. First, it was conducted at a single center, which may limit the generalizability of the findings. Second, the cross-sectional design prevents establishing a causal relationship between coronary microvascular dysfunction, stroke risk, cognitive decline, and metabolic biomarkers. Third, coronary microvascular dysfunction was assessed using available clinical and cardiovascular evaluations rather than standardized advanced methods such as coronary flow reserve measurement, PET, cardiac MRI perfusion imaging, or invasive coronary function testing, which may affect diagnostic precision. Fourth, stroke risk was categorized using clinical and vascular risk factors without a validated scoring system, limiting reproducibility. Fifth, cognitive decline was assessed through general clinical neurological evaluation rather than validated tools such as MoCA or MMSE, which may underestimate subtle impairment. Finally, only univariate analysis was performed, and potential confounders such as age, diabetes duration, hypertension, dyslipidemia, HbA1c, and LDL cholesterol were not adjusted through multivariate analysis.

## Conclusions

Coronary microvascular dysfunction was common among patients with type 2 diabetes mellitus without major vessel occlusion and was significantly associated with cognitive decline, elevated stroke risk, poor glycemic control, dyslipidemia, and hypertension. These findings suggest that coronary microvascular dysfunction may serve as a marker of systemic vascular impairment in diabetic patients. However, due to the cross-sectional nature of the study, causal relationships cannot be established. Further prospective longitudinal studies using standardized assessments of coronary microvascular dysfunction, validated cognitive evaluation tools, and comprehensive cerebrovascular risk stratification are needed to better understand these associations and their clinical implications.
